# Limitation and Potential Effects of Different Levels of Aging Corn on Performance, Antioxidative Capacity, Intestinal Health, and Microbiota in Broiler Chickens

**DOI:** 10.3390/ani11102832

**Published:** 2021-09-28

**Authors:** Ahsan Mustafa, Shiping Bai, Qiufeng Zeng, Xuemei Ding, Jianping Wang, Yue Xuan, Zhuowei Su, Keying Zhang

**Affiliations:** Institute of Animal Nutrition, Key Laboratory for Animal Disease-Resistance Nutrition of China, Ministry of Education, Sichuan Agricultural University, Chengdu 611130, China; dr.ahsan.mustafa@gmail.com (A.M.); shipingbai@sicau.edu.cn (S.B.); zqf@sicau.edu.cn (Q.Z.); dingxuemei0306@163.com (X.D.); wangjianping@sicau.edu.cn (J.W.); xuanyuede1007@hotmail.com (Y.X.); wzs698@126.com (Z.S.)

**Keywords:** aging corn, oxidative stress, broiler performance, intestinal health, microbiota

## Abstract

**Simple Summary:**

Corn is an important ingredient and staple food in China; thus, corn storage has a certain importance to ensure domestic food resources. Normally, corn has been stored for 3 or more years under the proper storage conditions in national barns before it is used as a feed ingredient. This study aimed to investigate the effect of different levels of aging corn (AC) on performance, antioxidative capacity, intestinal health, and microbiota in broilers. In the present study, AC grains were stored for 4 years under the proper storage conditions at the national storage facility. The results indicated that a lower level of AC diet showed improved performance and overall bird health than a higher level of AC, and comparable with a normal corn diet. However, antioxidative capacity is reduced by AC diets.

**Abstract:**

Three-hundred and sixty-day-old male broilers underwent three treatments with six replicates of 20 birds per treatment. The experimental diets included NC: normal corn diet; ACL: lower level (39.6–41.24%) of AC; and ACH: a higher level (56.99–59.12%) of AC. During phase 1 (0–21 d), broilers fed on AC showed lower (*p* < 0.05) body weight (BW), body weight gain (BWG), and feed conversion ratio (FCR) as compared with the NC group. During phase 2 (22–42 d), the NC group and ACL group showed better (*p* < 0.05) BW, BWG, and FCR than the ACH group. The footpad lesion score (*p* = 0.05) and litter moisture percentage (*p* < 0.05) were found to be higher in the ACH group. During phase 1, the ACL group showed a lower level of malondialdehyde (MDA) contents (*p* < 0.05) in serum; moreover, catalase (CAT) (*p* < 0.05) and glutathione peroxidase (GSH-Px) activities (*p* < 0.05) were found lower in both AC-containing groups. During phase 2, CAT activity in serum was found higher (*p* < 0.05) in the ACH group. During phase 1, the NC group showed higher CAT (*p* = 0.05), GSH-Px (*p* < 0.05), and superoxide dismutase (SOD) activity (*p* = 0.03); however, it showed lower MDA (*p* < 0.05) and total-antioxidative capability (T-AOC) (*p* < 0.05) in the liver. During phase 1, in breast muscle, CAT, SOD, and T-AOC were higher (*p* < 0.05) in the NC group. During phase 1, total cholesterol and high-density lipoprotein were found to be lower (*p* < 0.05) in the ACL group. Similarly, triglyceride and low-density lipoprotein were found to be lower (*p* < 0.05) in the ACL group than the ACH group. During phase 1, villus height was found to be higher (*p* < 0.05) in the ACH group. Moreover, the goblet cell (GC) was found to be higher (*p* < 0.05) in the NC group than the ACL group. During phase 2, GC was found to be higher (*p* < 0.05) in the ACL group. In ileal digesta, during phase 1, acetic acid, propionic acid, and butyric acid (BA) levels were found to be higher (*p* < 0.05) in the ACL group. In cecal digesta, BA was significantly lower (*p* < 0.05) in the NC group.

## 1. Introduction

Corn contains rich nutrients, including carbohydrates, water, vitamins, fiber, and minerals [1], and plays an essential role in China’s food crops. China is a major corn producer with more than 300 million acres of corn and is the second largest corn producer in the world [2]. A large number of corn has been stored as the country’s grain reserves to deal with unexpected natural disasters, and therefore the storage corn may be used as an animal feed when it was not suitable to store further [3]. It is expected that currently there are 200 million tons of stored corn, which could be stored for 3 to 4 years in China. During the storage time, some physiological and biochemical reactions happened in corn that may result in the loss of the nutritional value and accumulation of oxidation products in corn [4,5,6]. Ensuring the efficient use of stored corn in livestock and poultry, feed production is of great significance to the timely absorption of stock corn [7].

During storage, alpha- (α-) and beta- (β-) amylase enzyme activities could be decreased; however, lipoxygenase, lipases, and proteases enzyme activities of stored rice are increased [8,9]. Therefore, storage could reduce the digestibility and solubility of protein [9,10]. Moreover, Galliard [11] reported higher contents of free fatty acids (FFA) and oxidation of lipids in whole flour during storage, and Salman and Copeland [12] found an inversely proportional relationship between increased acidity of fat contents with decreased iodine binding capacity in wheat. The production of FFA in corn was found to be interlinked with the higher H_2_O_2_ production, which ultimately affects the activities of peroxidase (POD) and catalase (CAT) [13]. The lipid peroxidation of the cell membrane in stored grains could also alter the POC and CAT activities, which have been used as an indicator to evaluate the corn quality after storage [14].

The oxidation process and production of peroxides in aging corn, due to storage, can cause oxidative stress, which might be a cause of extreme oxidation inside the body tissues and create extremely reactive molecules, i.e., reactive species of nitrogen and radical of oxygen [15]. The free radicals can cause oxidative stress by the mean of a sequence of synthetic reactions comprising immunity, biochemistry, and physiology that ultimately affect the overall health and performance of livestock [16]. Likewise, the aging corn can take part in the production of resistant starch due to the degradation of starch during storage [17]. In the corn storage process, due to improper storage conditions, there are chances of mycotoxin contents, besides the alterations in corn’s chemical and physical properties, which are considered as a main cause of nutrients damage and limit the usage of aged corn [18]. Nutrients in corn expose to oxidation (such as oxidation of fatty acids) and corresponding activity (such as antioxidant enzymes) appears lower when these changes to a certain extent led to the generation of aging corn (AC).

It is reported that AC used as a feed ingredient in animals shows a negative impact on their body and health. These negative effects can be addressed and improved. Rational use and animal nutrition research is increasingly focusing on these issues. Studies have found that feed peroxidation destroys the redox balance of animals and leads to oxidative stress. Oxidative stress affects animal health, performance, reduced the FI and FCR, causes animal destruction of the intestinal integrity of the mucosa and barrier function of the intestine [19,20]. Lipid oxidation and FFA contents of AC are a major reason for poor performance and decrease serum oxidative capacity in broilers [3,21], laying hens [22], ducks [23], and weaned piglets [24], whereas Zhou et al. reported no adverse effect on the productive performance of laying hens [25]. The data found above suggest that the storage of corn under suitable conditions could not modify the nutritive value of corn. Contrary, the storage length had a harmful effect on the content of energy due to the increased FFA and decreased fat content [26]. However, to our best knowledge, there are no reports about the effect of AC levels on broilers’ health. Therefore, the present study was designed to investigate the effect of the higher and lower level of 4-year-old stored corn on the performance, antioxidative capacity, intestinal health, and microbiota in broilers.

## 2. Materials and Methods

### 2.1. Birds, Diets, and Management

The present study was accomplished by following guidelines of the standard recommendations of the National Institutes of Health for the Care and Use of Laboratory Animals. The current study protocol was approved by the Animal Care and Use Committee of Sichuan Agricultural University, China (Ethic Approval Code: SICAUAC201710-7).

Three-hundred and sixty-day-old male broiler chicks (Ross 308) were obtained from a local commercial hatchery (Yuguan Co. Ltd., Chengdu, China) and reared in a broiler house, which was environmentally controlled, on floor pens (dimension of 6.56 × 3.28 ft.), with a litter of rice husk at broiler experimental farms, Sichuan Agricultural University, Ya’an, China. This experiment was a completely randomized block study and comprises of 3 treatments with 6 replicates of 20 birds per treatment. Broilers were randomly assigned to the treatments with two phase-feeding programs (Phase 1: Day 1–21, Phase 2: Day 22–42). The basal diet was formulated as a corn–wheat–soybean-based diet according to the NRC [27] nutrient recommendations (Table 1). Experimental diet comprises as follows; NC: control diet with normal corn; ACL: control diet with a lower level (39.6–41.24%) of aging corn; ACH: control diet with a higher level (56.99–59.12%) of aging corn. All the diets were processed in pellet (3 mm) form. Feed and water were provided to broilers ad libitum. Bird management was conducted as described in the Ross 308 Broiler Commercial Management Guide.

### 2.2. Aging Corn

Normal corn and AC originated from the national barns in Yinchuan, China, and Changchun, China, which had been stored for 6 months and 4 years, respectively. All corn samples were stored in brick structures, and some phytochemical properties were determined (Table 2). According to the methods described by AOAC International [28], the dry matter (DM), crude protein (CP), and crude fiber (CF) were analyzed by oven drying (Method No. 934.01), Kjeldahl (Method No. 990.3), and Soxhlet fat analysis (Method No. 920.39), respectively. Gross energy was analyzed by adiabatic bomb calorimetry (Parr Instrument Company, Moline, IL, USA). The quantity of potassium hydroxide (KOH) required for acid neutralization in a 100 g sample was used for the analysis of titratable acidity (GB/T 20570-2015). The POD and CAT activity, as well as malondialdehyde (MDA) contents, were measured by using precise detection kits from Nanjing Jiancheng Bioengineering Institute (Nanjing, China) with a Multiskan Spectrum Reader (Model 1500; Thermo Scientific, Nyon, Switzerland). Deoxynivalenol, zearalenone, and aflatoxin were measured by using the national standard methods, i.e., SN/T 1571-2005, GB/T 28716-2012, and GB/T 30955-2014, respectively. Similarly, the standard method for fatty acids (FAs) was used for its quantification (GB 5009.168-2016).

### 2.3. Growth Performance

Individual broilers, after 12 h fasting, body weight (BW), and the amount of feed intake (FI; offered–refused) by pen were measured at the end of each phase (1 and 2) at 21 and 42 d. Then, average body weight (ABW), average body weight gain (ABWG), average feed intake (AFI), and feed conversion ratio (FCR) by pen were calculated. Birds were monitored to account for their morbidity (health status) and mortality on a daily basis. During the experiment, the BW of dead birds was included in growth performance calculations.

### 2.4. Footpad Dermatitis Score

Footpad dermatitis scoring was conducted at the end of phase 1 and phase 2 by visual inspection of the footpad of both feet of all live birds in each pen. A seven-point scoring system was followed, according to the procedure of Mayne et al. [29]. The better quality of the footpad is associated with a lower footpad dermatitis score. The total footpad score (*TFPS*) for each pen was calculated as follows.

(1) $$TFPS=\frac{SUM\;OF\;AVERAGE\;OF\;2\;FEET\;FOOTPAD\;SCORE}{TOTAL\;NUMBER\;OF\;BIRDS\;EXAMINED\;IN\;EACH\;PEN\;}$$

### 2.5. Litter Moisture Percentage

**Figure 1:**
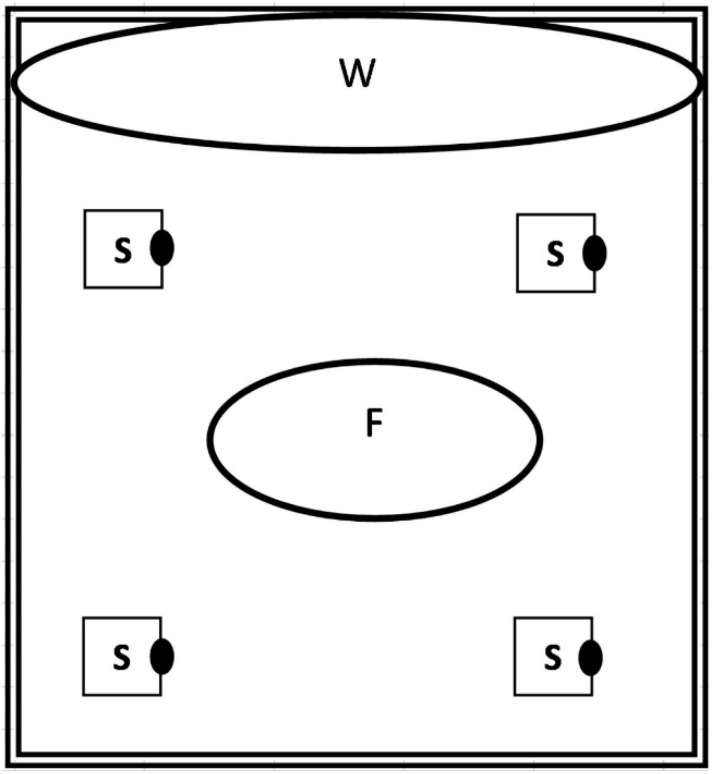
Litter sampling in each pen. Waterer (W), feeder (F), and sampling location (S).

Litter samples were taken from each pen at the end of phase 1 and phase 2. Four sub-samples were taken from each pen from the surface to the full depth of the accumulation at locations shown in Figure 1. Sub-samples were taken from the middle of each side except the side with the door, avoiding the drinker and feeders. Side sub-samples were taken approximately 30 cm from the wall at the side of the pen. The four sub-samples were mixed per pen for analysis for moisture. Litter moisture was determined by placing litter samples in a forced-air drying oven at 60 °C for more than 24 h.

### 2.6. Sample Collections

After phases 1 and 2, two birds from each replicate (12 broilers from each treatment), with BW near to the average BW of the pen, were selected and slaughtered by severing their jugular vein. Before slaughtering, the samples of blood were collected through the jugular vein, and afterward, for serum collection, these blood samples were centrifuged (2000× *g*, 10 min, 4 °C). The collected serum was then stored at −20 °C for further biochemical parameter analysis. After serum was collected from 12 birds, from them, 6 birds were slaughtered by severing their jugular vein. Liver tissue, breast muscle, and gastrointestinal tract (GIT) samples were collected. Serum, liver, and breast muscle samples were stored at −20 °C for antioxidant activity analysis. The GIT was immediately dissected after euthanization, and a half portion of jejunum was stored in 4% paraformaldehyde solution of histological analysis. However, another half portion of jejunum was used for jejunum mucosa flash-frozen in liquid nitrogen −80 °C until gene expression analysis. Moreover, from the same birds, ileal and cecal digesta samples were collected by squeezing them gently and stored at −20 °C for short-chain fatty acid (SCFA) analysis. On days 21 and 42, the remaining six birds were slaughtered to collect cecal digesta and this was stored at −80 °C for microbial community analysis.

### 2.7. Antioxidant Activity

Six serum, liver, and breast muscle samples from each treatment were used to analyze MDA content, the enzymatic activity of CAT, superoxide dismutase (SOD), glutathione peroxidase (GSH-Px), and the total antioxidative capability (T-AOC) using commercial assay kits (Nanjing Jiancheng Institute, Nanjing, China) and procedure described previously by Che et al. [30].

### 2.8. Serum Profile

Six serum samples from each treatment were used to analyze triglyceride (TG), total cholesterol (TC), high-density lipoprotein (HDL), and low-density lipoprotein (LDL) concentrations, as well as alanine aminotransferase (ALT) and aspartate aminotransferase (AST) activities, which were important indices to evaluate lipid metabolism and liver function, using an automatic biochemistry analyzer Hitachi 7020 (Hitachi High Technologies Inc., Tokyo, Japan) with kits being purchased from the Nanjing Jiancheng Bioengineering Research Institute, Nanjing, China.

### 2.9. Intestinal Characteristics

#### 2.9.1. Jejunum Morphology and Counting of Goblet Cell

Fixed segments in 4% neutral buffered paraformaldehyde solution were rinsed in ethyl alcohol and embedded in paraffin wax. The samples were cut (5 µm) using a propeller slicer (Leica-2016, Leica Inc., Bensheim, Germany), with 3 slices per treatment, and stained using the hematoxylin and eosin method. The micrographs were taken using a microscope (BA400Digital, Motic China Group Co. Ltd., Xiamen, China) was used to take micrograph, and measurements were performed for villus height (VH), crypt depth (CD), and calculated ratio of villus height to crypt depth (VH:CD), as well as count the goblet cells (GC) and calculate the number of goblet cells per unit area by using Image-Pro Plus 6.0 (Media Cybernetics, Rockville, MD, USA) for each structure per slice. The tip of the villus to the villus-crypt junction was defined as VH, whereas the CD was measured from the depth of the invagination to adjacent villi [31].

#### 2.9.2. Jejunum Mucosal mRNA Gene Expression

After phase I and II, from jejunal mucosa, total RNA was extracted by using a TRIzol reagent kit (Takara, Dalian, China), and synthesis of cDNA was completed by using the reagent kit (PrimeScript RT, Takara, Kusatsu, Japan). Primers for Tight Junction Protein (TJP), 3 genes in association with the intestinal barrier including Claudin1 (CLDN1), Zonula Occludens-1 (ZO-1), Occludin (OCLN), and β-actin (housekeeping gene) were designed using Primer Express 3.0 (Applied Biosystems, Waltham, MA, USA; Table 3). Real-time quantitative PCR was performed according to Livak and Schmittgen, [32].

### 2.10. Ileal and Cecal Digesta Analysis

#### 2.10.1. Volatile Fatty Acid Analysis

At 21 and 42 d, proportions of the volatile fatty acid (VFA), i.e., acetic acid (AA), propionic acid (PA), and butyric acid (BA), were determined from ileal and cecal digesta by using the HPLC system following the method Qin et al. [35] with some modifications. Approximately 0.5 g ileal and cecal contents were gently transferred into a micro-centrifuge tube containing 2 ml of ultrapure water. The solution was thoroughly mixed using a vortex mixer and centrifuged at 5000 rpm for 10 min at 4 °C. Taking 1 mL supernatant added 0.2 mL ice-cold 25% (*w*/*v*) meta-phosphoric acid solution, incubated at 4 °C for 30 min, and again centrifuged @12,000 rpm for 10 min. The supernatant was filtered through 0.22 µm syringe filters. The VFA contents of this filtrate were measured using a gas chromatograph (CP-3800, Varian, Palo Alto, CA, USA).

#### 2.10.2. 16S rDNA Gene Amplicons Analysis

At d 21 and 42, cecal digesta samples were subjected for extraction of DNA by using QIAamp PowerFecal DNA Kit (Qiagen, Hilden, Germany) according to the manufacturer’s instructions. The DNA concentration and quality were checked using a NanoDrop Spectrophotometer. The Novo gene platform (Illumina Hiseq, Novogene Bioinformation Technology, Beijing, China) was used to performed 16S rDNA gene amplicons analysis. All methods including extraction of DNA, 16S rRNA sequencing, processing of sequences, and analysis of data were performed according to Qin et al. [36]. Concisely, sterile water was used to dilute DNA up to 10 ng/μL. The 16S rRNA genes of distinct regions (16S V4) were amplified using a specific primer (515F GTGCCAGCMGCCGCGGTAA; 806R GGACTACHVGGGTWTCTAAT) with the unique barcodes. The Phusion High-Fidelity PCR Master Mix (New England Biolabs) was used for the accomplishment of all PCR reactions. The end products of PCR were mixed in equal density ratios. Then, Qiagen Gel Extraction Kit (Qiagen) was used for purification of these PCR mixture products. Sequencing libraries were generated using TruSeq DNA PCR-Free Sample Preparation Kit (Illumina, San Diego, CA, USA) following the manufacturer’s recommendations, and index codes were added. The Agilent Bioanalyser 2100 system and the Qubit 2.0 Fluorometer (Thermo Scientific) were used to assess the quality of these libraries. Lastly, the sequencing of eligible libraries was completed on an Illumina HiSeq 2500 platform and generated the 250 bp paired-end reads. QIIME quality filters were used for the filtration of selected reads. Sequences with ≥97% similarity were assigned to the same optimal taxonomic units (OTUs). The relative abundance of each OTU was examined at different taxonomic levels. Diversity within communities (Alpha diversity) calculations and taxonomic community assessments were performed by Qiime 1.7.0, and Beta diversity included both unweighted and weighted Unifrac distances calculated with 10 times subsampling; distances were visualized by principal component analysis (PCA; Lozupone and Knight) [37], and the separation was tested using R in Anosim.

### 2.11. Statistical Analysis

The experiment was a completely randomized design with a pen as the experimental unit. For serum parameter, antioxidative parameters, intestinal characteristics, short-chain fatty acids, and diversity and structure of the cecal microbiota analysis, the randomly selected birds were the experimental unit, and results were analyzed by a one-way analysis of variance (ANOVA) using the GLM procedure of SAS (SAS Institute Inc., Cary, NC, USA). Differences among means were tested with Duncan’s multiple range tests. *p* ≤ 0.05 is considered significant.

## 3. Results

### 3.1. Growth Performance

The growth performance responses of the broiler chickens under different experimental groups are shown in Table 4. During phase 1, the ACL group has lowered (*p* < 0.05) ABW and AWBG from NC and ACH groups, whereas ACH groups have lowered (*p* < 0.05) ABW and AWBG from the NC group, and similarly FCR was poor (*p* < 0.05) in ACL group from NC diet, and numerically poor from the ACH group. On 42 d, ABW was significantly (*p* < 0.05) and numerically lower in the ACH group from the NC group and ACL group, respectively. Similarly, during phase 2 and the whole experiment (1–42 d), AWBG and FCR were found to be poor (*p* < 0.05) in the ACH group among all dietary treatments. The FI and mortality were not significantly different among treatments during phase 1, phase 2, and during the whole experiment (1–42 d). Overall, during the whole experimental period, a lower level (39.6–41.24%) of AC has shown comparable performance with normal corn, whereas a high level (56.99–59.12%) of AC has shown lower performance than other dietary treatments.

### 3.2. Footpad Dermatitis Score and Litter Quality

Both footpads were gross examined on the d 21 and d 42 for allotting the proper score according to their pathological condition (Table 5). A similar trend was observed in TFPS between experimental groups after phase 1 and phase 2. After 21 d, TFPS was significantly lower (*p* = 0.05) in the ACL group from the ACH group and numerically lower from the NC group. Similarly, after 42 d, TFPS in the ACL group was numerically lower and higher from ACH and NC groups, respectively. Litter moisture percentage (%) was also measured on 21 d and 42 d (Table 5) and found similar results as TFPS. On d 21, litter moisture was numerically lower in the ACL from NC and ACH groups. After 42, the litter moisture percentage was significantly higher (*p* < 0.05) in the ACH group than in the NC and ACL groups. Convincingly, a higher level (56.99–59.12%) of AC in the diet caused adverse effects on footpad dermatitis score and litter moisture as compared with normal corn and lower levels (39.6–41.24%) of AC.

### 3.3. Antioxidant Activity

The enzymatic activity of CAT, SOD, GSH-Px, T-AOC, and MDA contents were measured in serum, breast muscle, and liver on d 21 and 42 (Table 6). On d 21, CAT (*p* < 0.05) and GSH-Px (*p* < 0.05) were found higher in the NC group than ACL and ACH groups, and MDA was found lower (*p* < 0.05) in the ACL group than NC and ACH groups in serum; however, SOD and T-AOC were not significantly different among all treatment groups. For the liver, the NC group shows higher CAT (*p* = 0.05) than the ACH group, and higher GSH-Px (*p* < 0.05) than ACL and ACH groups, similarly SOD was found higher (*p* < 0.05) in the NC group than ACL group; however, MDA found lower (*p* < 0.05) in the NC group from all other treatment, and TAOC also found lower (*p* = 0.05) in the NC group than the ACL group. For breast muscles, CAT was higher (*p* < 0.05) in the NC group than the ACH group, and SOD and TAOC were found higher (*p* < 0.05) in the NC group than all other treatment groups. However, the lowest TAOC was found in the ACH group. Moreover, GSH-Px and MDA were found to be similar among all the treatment groups.

On d 42, CAT was found to be higher (*p* < 0.05) in the ACH group from other treatment groups; however, T-AOC was found higher (*p* < 0.05) in the NC group than ACL group in serum, whereas GSH-Px, SOD, and MDA were found similar among all treatment groups in serum. However, for the liver, all antioxidant indices were found to be similar among all dietary treatments. For breast muscle, CAT was found to be higher (*p* < 0.05) in the NC group than other treatment groups, and SOD was found to be higher (*p* < 0.05) in the ACH group than other treatment groups. Other indices including GSH-Px, MDA, and TAOC were found to be similar among all treatment groups.

### 3.4. Serum Profile

Results of important indices to evaluate liver function (AST and ALT) and lipid metabolism (TC, TG, HDL-C, and LDL-C) through serum analysis during both phases 1 and 2 are shown in Table 7. During phase 1, both AST and ALT were found to be similar among all dietary treatments. However, TC and HDL-C were found to be lower (*p* < 0.05) in the ACL group than NC and ACH groups. Similarly, TG and LDL-C were found to be lower (*p* < 0.05) in the ACL group from the ACH group. Although during phase 2, serum levels of AST, ALT, TC, TG, HDL-C, and LDL-C were found to be non-significantly different from each other, the ACL group has a lower value for all serum indices.

### 3.5. Intestinal Morphology and mRNA Gene Expression

**Figure 2:**
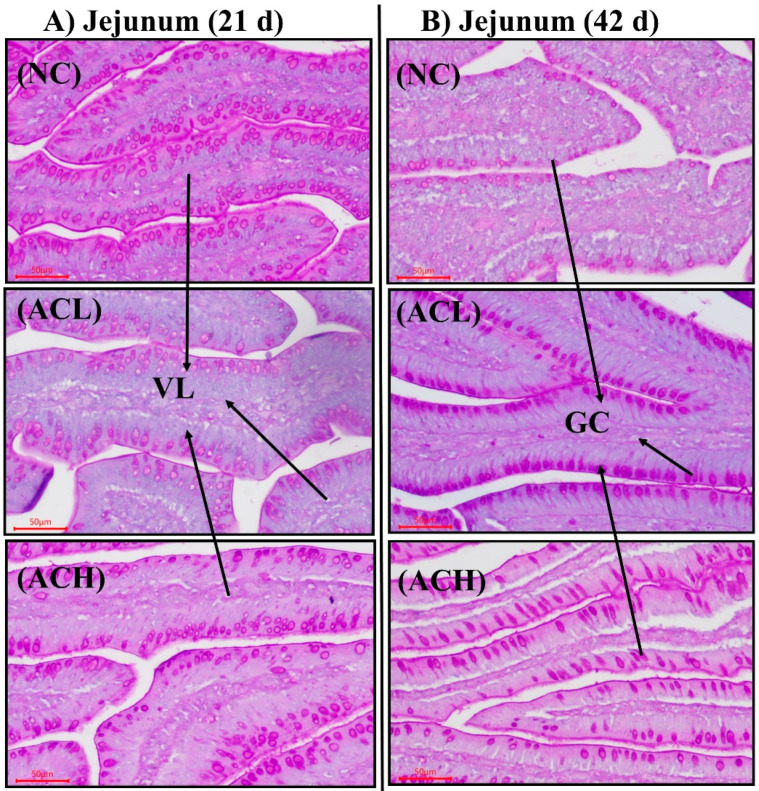
Jejunum villus morphology. Intestinal specimens (*n* = 6) were collected at 21 d (**A**) and 42 d (**B**); normal corn diet (NC); low aging corn (ACL); high aging corn (ACH). VH = villus height; GC = goblet cell.

On day 21, jejunal morphological results were observed to be higher VH in the ACH group from NC and ACL group (*p* < 0.05) shown in Table 8. Similarly, VH:CD was also found to be higher (*p* < 0.05) in the ACH group from the NC group, whereas CD was found to be similar among the dietary treatments. Moreover, the GC was found to be higher in the NC group (*p* < 0.05; Table 8; Figure 2) than in the ACL group. On day 42, VH:CD was found higher in the NC group than ACL and ACH groups (*p* < 0.05), and GC was found higher in the ACL group than NC and ACH groups (*p* < 0.05), whereas VH and CD were found to be similar among all the treatments. Jejunal mRNA gene expression for TJP results (Table 8) indicated that CLDN1, ZO-1, and OCLN were not significant among all dietary treatments during both phases 1 and 2, whereas the ACL group has shown more improved results than the ACH group, and these results are comparable with the NC group.

### 3.6. Ileal and Cecal VFA Contents

In ileal digesta, after phase I, AA level was higher (*p* < 0.05) in the ACL group than NC group and similar with the ACH group; moreover, PA and BA levels were found significantly (*p* < 0.05) higher in the ACL group from all other treatment groups (Table 9). After phase II, AA and PA were found similar among all the experimental groups; however, BA was significantly lower in the NC group than all other treatment groups (*p* < 0.05). In cecal digesta, AA and PA were found to be similar among all the experimental groups; however, BA was significantly lower in the NC group than the ACH treatment group (*p* < 0.05) after phase I, whereas after phase II, PA and BA were found to be lower in the NC group than the ACL group (*p* < 0.05) and similar to the ACH group. The level of AA was found to be similar among all experimental groups.

### 3.7. Cecal Microbial Community

**Figure 3:**
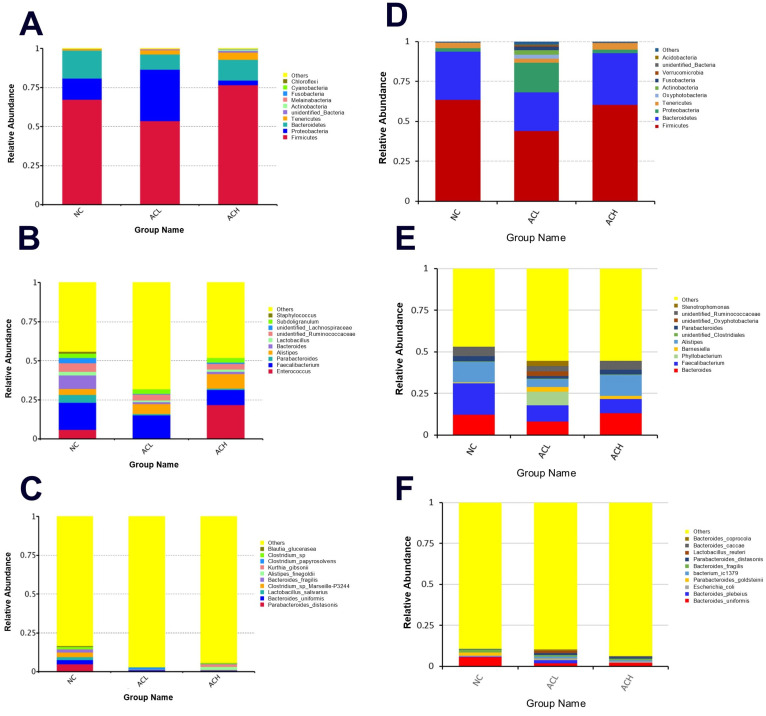
Taxonomic differences in the microbial community of the caeca in broilers (*n* = 6) on 21 d with relative abundance levels of the bacterial (**A**) phyla, (**B**) genu, and their (**C**) species, and on 42 d with relative abundance levels of the bacterial (**D**) phyla, (**E**) genu, and their (**F**) species.

The microbial communities were compared in the cecum among five dietary groups, using Illumina Hiseq high-throughput sequencing. On day 21, a total of 1,083,368 sequencing reads were obtained from the cecal digesta samples, and through cutting and filtering of reads, an average of 83,336 reads was measured per sample, and an average of 78,125 valid data was obtained after quality control. The effective rate of quality control was 93.8%. The sequences were clustered into OTUs (operational taxonomic units) with 97% identity. At the phylum level, the most dominant species were *Firmicutes*, *Proteobacteria*, and *Bacteroidetes*, and their abundance was not significantly different among all experimental groups (Figure 3A). At the genus level, dominant but significantly similar, the species present were *Enterococcus*, *Faecalibacterium*, and *Parabacteroides* (Figure 3B). Similarly, at the species level, the dominant species were *Parabacteroides distasonis*, *Bacteroides uniformis*, and *Lactobacillus salivarius*, and were found to be similar among the all-treatment groups. On day 42, a total of 1,546,555 sequencing reads were obtained from the cecal digesta samples, and through cutting and filtering of reads, an average of 85,919 reads was measured per sample, and an average of 81,499 valid data was obtained after quality control. The effective rate of quality control was 94.8%. The sequences were clustered into OTUs (operational taxonomic units) with 97% identity, and a total of 3110 OTUs were obtained. At the phylum level, the most dominant species were *Firmicutes*, *Bacteroidetes,* and *Proteobacteria,* and their abundance was not significantly different among all experimental groups (Figure 3D), whereas *Fusobacteria* (*p* < 0.05) was significantly higher in the ACL group as compared with all other treatments. At the genus level, the dominant species were *Bacteroides*, *Faecalibacterium*, and *Phyllobacterium*, and Bacteroides and *Phyllobacterium* were found to be statistically similar, whereas *Faecalibacterium* was significantly higher (*p* < 0.05) in the NC group than other dietary treatments (Figure 3E). At the species level, the dominant species were *Bacteroides uniformis*, *Bacteroides plebeius*, and *E. Coli*, and found to be similar among all treatment groups. Moreover, all other species were found to be non-significantly different from each other (Figure 3F).

After phase 1, the result of alpha (α) diversity including observed species (OS), Shannon (SH), Simpson (SI), Chao1 (CH), abundance-based coverage estimator metric (ace), good coverage (gc), and phylogenetic distance (PD) were found to be similar among all dietary treatments (Table 10). However, after phase 2, the result of all parameters of α-diversity except PD was found similar among all experimental groups (Table 10). PD was observed to be higher (*p* < 0.05) in the ACL group (Table 10). Additionally, the relationships between communities of various bacteria belonging to different treatments were characterized by PCA, and the results exhibited that there was no significant difference among microbial communities of cecal digesta from different experimental groups during both phase 1 and phase 2.

## 4. Discussion

Various studies have designated storage conditions and time had adverse effects on cereal grains, specifically their chemical composition [6,38,39], as storage time is directly proportional to the acidity value of stored corn [40]. The results of the present study corroborated earlier findings that AC fat acidity was found to be higher when compared with corn that had been stored for a few months. These findings indicated that prolonged storage is not suitable for AC. Moreover, others scientists reported that lipid present in AC can be oxidized and formed hydroperoxides (H_2_O_2_) [41], and CAT and POD were decreased during grain storage [3]. These findings can be used as an indicator of cereal grain deterioration during storage, i.e., rice [42]. The AC quality evaluation mainly depends on the POD activity, and FAs’ value includes the important parameters of the quality evaluation of corn storage. In the present study, after chemical evaluation of AC, it was observed that the FAs’ value and POD activity of AC were found to be higher and lower, respectively, than normal corn. The AC came from the grain national depot, therefore the storage conditions were suitable, and the content of mycotoxins was not considerable, thus the adverse effects of the AC-containing diet on broilers were caused by changes in the corn nutritional components, such as FA oxidation.

In the present study, the performance was affected during both the starter phase and growing phase in broilers fed on the lower and higher level of AC, respectively. The BW and BWG were found to be highest in the normal corn diet, followed by a higher AC level diet, and the lowest was found in a lower AC level diet. The higher AC level diet showed higher BW and BWG than the lower AC level diet during the starter period because it contains a higher level of soybean, as this diet is based on corn–soybean, whereas other diets based on corn–wheat–soybean diets, and soybean meal is a major source of protein due to its higher digestibility and amino acid availability [43]. However, during the growing phase, a higher AC diet attained the lowest BW and BWG, as well as having poorer FCR than the other groups. Interestingly, a lower level of AC diet shows improved FCR even from the normal corn diet. That indicated the positive effect of the lower level of AC as compared with a higher level of AC. A similar result was reported in ducks as a higher level of AC produced a more adverse effect on duck health as compared with a lower level of AC [23]. Another report in layers [25] as AC produced no negative effect on production performance. The performance results are interlinked with intestinal morphology as it was found to be balanced during the starter phase in broilers fed on a higher level of AC, whereas during the growing phase, it was found improved in broilers fed on the lower level of AC. As improved intestinal morphology can enhance digestibility, this ultimately improves performance in birds [44]. Therefore, it can be assumed that a lower level of AC could enhance digestibility and reduce the water content in excreta, as high moisture in excreta is a major cause of footpad lesions in broilers [45]. Improved intestinal health and digestibility could be a reason for lower litter moisture and footpad score in broilers fed on lower level of AC as compared with a higher level of AC.

The antioxidant capacity was found to be lower in serum, liver, and breast muscle by the inclusion of both higher and lower quantities of AC during the starter and growing period. These results indicating that the storage of corn can decrease the activity of antioxidant enzymes due to the presence of FFA, because FFA oxidized easily to produce H_2_O_2_, and these can affect the activities of enzymes such as POD and CAT in maize [13]. Similar results were reported as old corn lowers the serum antioxidative capacity in broilers [46]. Under the condition of oxidative stress, the activities of T-AOC, GSH-Px, CAT, and SOD in the serum were decreased; however, the MDA contents were increased [43]. Long-term corn storage could cause a negative impact on birds’ health. Ducks fed AC diets were more likely to obtain oxidative damage, which resulted in reduced growth performance [23,47]. Moreover, MDA in serum and liver was found higher with a higher level of AC in the diet. MDA is a lipid peroxidation (LPO) and the main product of degradation, which reflects the lipid peroxidation (damage) in the body, and cells are attacked by free radicals. The MDA content was also found to be higher in stored corn, which can cause lipid peroxidation in broilers [21]. Liu et al. [46] also reported higher serum MDA content in broilers fed on the old corn.

In the growing animals, nutritional status is mainly reflected by their serum biochemical parameters [48]. Our results showed that a lower level of AC influences lipid metabolism during the starter phase. The lower TG in AC groups may be attained by lowering the FAs synthesis inside the liver and the activation of peroxisome proliferator activating receptor α, which enhances β-oxidation of FAs [49,50]. In this study, a lower level of AC significantly reduced various forms or types of cholesterol including TC, HDL-C, and LDL-C in serum. In another study, rats were fed on oxidized oil containing products of lipid peroxidation, and resulted in reducing cholesterol and triacylglycerols concentrations in the plasma and liver [51]. Likewise, Koch et al. [52] reported that oxidized oil significantly decreased the contents of VLDL-C, HDL-C, and TC in the liver and plasma. A similar effect of AC on serum lipid profile was reported in layer [22].

The villus inside the small intestine, i.e., the jejunum, is a major site for digestion of food and absorption of nutrients in monogastric animals. Consequently, the proper functioning of gut microbiota, immune system, and nutrition, could be achieved by a healthy mucosa of the small intestine. During the growing phase, a higher level of AC had adverse effects on intestinal morphology as compared with a lower level of AC and NC groups. Similar results have been reported by various scientists [47,53]. It can be assumed that lipid peroxidation in a higher level of AC might stimulate the jejunal mucosa and causes oxidative stress that ultimately increases the energy demand of birds as the free radicals damage the mucosa of the small intestine. The VH:CD is considered to play a key role during digestion and absorption. In the present study, a higher level of AC shows lower VH:CD in the jejunum indicated that AC in diets may damage the intestinal mucous to some extent and causes the reduction in the absorptive capacity. Moreover, intestinal morphology, tight junction proteins have also played key roles in maintaining the integrity of the intestinal barrier and regulating intestinal permeability [54]. In the present study, although no significant effect was observed between all dietary treatments, the ACL group has shown improve TJP than the ACH group.

In broilers, the major concentration of analyzed SCFAs of ileal and cecal chyme can be varied mainly due to the ingredients of chicken feed [55]. The present study provides novel information regarding ileal and cecal SCFAs in broilers fed on the higher and lower level of AC as it has never been tested previously. During both dietary phases, all SCFAs in ileum and cecum were found to be higher in broilers fed on a diet containing AC aging corn as compared with boilers fed on the diet containing normal corn. Mainly SCFAs are produced in the GIT due to the fermentation of complex carbohydrates [56]. Therefore, it could be assumed that AC might contain the complex carbohydrates that could be contributed to the production of SCFAs in the GIT of broilers. No study has been reported regarding the effect of AC on the intestinal microbial community in the broiler. Although microbiota including taxonomy, alpha diversity, and beta diversity were found to be similar among all the treatment and control groups, the SCFAs improved the intestinal health in broilers fed AC, especially the lower level of AC. Various researchers reported the positive effect of SCFAs on intestinal health in broilers [57] and pigs [58].

## 5. Conclusions

After phase 1, the higher level (56.9%) of AC had improved the performance compared with the lower level (39.6%); however, after phase 2, the higher level (59.12%) had more negative effects on performance and broilers’ health than lower level (41.24%). As the higher level (59.12%) showed lower performance, antioxidant capacity, and lipid profile, which interlinked with decreased intestinal health that causes more water in dropping and resulting in higher footpad necrosis. However, a lower level (41.24%) of AC diet showed a more improved performance than a higher level (59.12%) of AC and was comparable with a normal corn diet. However, antioxidative capacity was found to be lower in both AC groups. Better performance and overall bird health could be obtained from lower levels (39.6–41.24%) of AC in the broiler diet by using some antioxidants as a supplement in the broiler diet.

## Figures and Tables

**Table 1:** Formulation of basal diets ^1^ fed to broilers during Phases 1 and 2.

Ingredients (%)	Phase 1 (1 to 21 d)		Phase 2 (21 to 42 d)	
	NC/ACL	ACH	NC/ACL	ACH
Corn (Normal or Aging)	39.6	56.99	41.24	59.12
Wheat	20.1	0	21	0
Soybean Meal (43%)	33.76	36.7	30.74	34
Soybean Oil	2.82	2.6	3.53	3.4
Limestone (CaCO_3_)	1.05	1.1	0.86	0.93
Calcium hydrogen phosphate	1.4	1.4	1.33	1.33
Salt (NaCl)	0.4	0.4	0.4	0.4
Choline chloride	0.15	0.15	0.15	0.15
Multi-vitamins ^2^	0.03	0.03	0.03	0.03
Mineral premix ^3^	0.2	0.2	0.2	0.2
Lys HCl (99%)	0.24	0.18	0.27	0.2
DL-methionine (99%)	0.25	0.25	0.25	0.24
Total	100	100	100	100
Nutrient level (calculated)				
ME (kcal/kg)	2950	2950	3020	3020
CP (%)	21	21.03	20	20.07
Calcium (%)	1	1	0.9	0.9
Available phosphorus (%)	0.45	0.45	0.43	0.43
Lysine (%)	1.15	1.15	1.1	1.1
Methionine (%)	0.5	0.5	0.48	0.48
Methionine + Cysteine (%)	0.86	0.85	0.83	0.81

^1^ Normal corn diet (NC); low aging corn (ACL); high aging corn (ACH). ^2^ Vitamin premix per kilogram feed provided: Vitamin A, 16,000 IU (trans retinol); Vitamin D3, 4000 IU; Vitamin E, 1IU (dl-α-tocopheryl acetate); Vitamin B1, 0.8 mg; Vitamin B2, 6.4 mg; Vitamin B12, 0.012 mg; Vitamin B6, 2.4 mg; calcium pantothenate, 10 mg; niacin acid, 14 mg; biotin, 0.1 mg; folic acid, 0.2 mg; Vitamin K3, 2 mg. ^3^ Mineral premix per kilogram feed provided: Fe (FeSO_4_·H_2_O), 100 mg; Cu (CuSO_4_·5H_2_O), 12.5 mg; Mn (MnSO_4_·H_2_O), 88 mg; Zn (ZnSO_4_·H_2_O), 95 mg; I (KI), 0.9 mg; Se (Na_2_SeO_3_), 0.3 mg.

**Table 2:** Phytochemical properties of normal and aging corn (air-dried basis).

Items	Content	
	Normal Corn	Aging Corn
Gross energy (kcal/g)	3.80	3.85
Crude protein (%)	7.56	7.73
Moisture (%)	14.31	13.06
Crude fat (%)	3.42	3.31
ADF (%)	9.80	10.1
aNDF (%)	10.7	12.4
Xanthophylls (mg/kg)	15.5	4.9
Fatty acids acidity (KOH mg/100 g)	64	126
MDA (nmol/mL)	96.03	40.30
POD (U/mg)	64.93	34.26
CAT (U/mg)	28.49	17.00
Aflatoxin (μg/kg)	1.9	^†^
Vomitoxin (μg/kg)	^†^	240.9
Zeranol (μg/kg)	63.4	87.4
Fatty acids (mg/g)		
Palmitic (C16:0)	3.156	2.448
Stearic (C18:0)	0.314	0.256
Oleic (C18:1)	5.727	3.872
Linoleic (C18:2)	11.391	9.232
Linolenic (C18:3)	0.318	0.012

^†^ Non-detected element.

**Table 3:** Primers used for the quantitative RT-PCR of the target genes.

Target Gene	Forward/Reverse Sequence (5′ to 3′)	Gen Bank Accession No.	Reference
β-actin	F: TTGGTTTGTCAAGCAAGCGG	NM_205518.1	[33]
	R: CCCCCACATACTGGCACTTT		
Zonula Occludens-1	F: TGTAGCCACAGCAAGAGGTG	XM_413773	
	R: CTGGAATGGCTCCTTGTGGT		
Claudin-1	F: TGGAGGATGACCAGGTGAAGA	NM_001013611.2	[34]
	R: CGAGCCACTCTGTTGCCATA		
Occludin	F: TCATCGCCTCCATCGTCTAC	NM_205128.1	
	R: TCTTACTGCGCGTCTTCTGG		

**Table 4:** Effect of different levels of aging corn on performance parameters under the three experimental diets ^1^ in broilers.

Measurements	NC	ACL	ACH	SEM	*p*-Value
Average Body Weight (g)					
1 d	45.02	45.07	45.08	0.07	0.78
21 d	1000.2 ^a^	935.7 ^c^	972.1 ^b^	9.0	<0.0001
42 d	2732.0 ^a^	2692.0 ^ab^	2589.0 ^b^	37.4	0.032
Average Body Weight Gain (g)					
1–21 d	955.2 ^a^	890.6 ^c^	927.0 ^b^	9.0	<0.0001
22–42 d	1738.0 ^a^	1757.0 ^a^	1617.0 ^b^	34.2	0.028
1–42 d	2687.0 ^a^	2647.0 ^ab^	2543.0 ^b^	37.4	0.032
Average Feed Intake (g)					
1–21 d	1299.0	1261.0	1279.0	11.9	0.08
22–42 d	3680.0	3571.0	3644.0	64.3	0.46
1–42 d	4849.0	4741.0	4821.2	64.0	0.47
Feed Conversion Ratio (g/g)					
1–21 d	1.36 ^b^	1.42 ^a^	1.38 ^ab^	0.01	0.009
22–42 d	2.13 ^ab^	2.03 ^b^	2.24 ^a^	0.04	0.014
1–42 d	1.81 ^b^	1.79 ^b^	1.88 ^a^	0.02	0.028
Mortality (%)					
1–21 d	3.33	2.50	0.83	1.14	0.28
22–42 d	7.77	10.58	3.82	2.42	0.24
1–42 d	10.00	11.66	4.17	2.54	0.16

^1^ Normal corn diet (NC); low aging corn (ACL); high aging corn (ACH). The data are presented as the mean of *n* = 6 for each group. ^a–c^ Mean within each column with no common superscript differ significantly (*p* < 0.05).

**Table 5:** Effect of different levels of aging corn on litter moisture and foot-pad dermatitis score in the broiler.

Treatments ^1^	Footpad Score		Litter Moisture%	
	Phase I	Phase II	Phase I	Phase II
NC	1.69 ^ab^	4.17	34.60	51.10 ^b^
ACL	1.46 ^b^	4.63	31.50	51.50 ^b^
ACH	2.44 ^a^	5.35	33.93	55.63 ^a^
SEM	0.26	0.53	1.57	1.28
*p*-value	0.050	0.271	0.345	0.044

^1^ Normal corn diet (NC); low aging corn (ACL); high aging corn (ACH). The data are presented as the mean of *n* = 6 for each group. ^a,b^ Mean within each column with no common superscript differ significantly (*p* < 0.05).

**Table 6:** Effect of different levels of aging corn on the oxidative ability of serum, liver, and breast muscle in broilers.

Measurements ^2^		Treatments ^1^				
		NC	ACL	ACH	SEM	*p*-Value
		Phase I (21 d)				
Serum	CAT (U/mL)	0.80 ^a^	0.63 ^b^	0.56 ^b^	0.05	0.005
	GSH-Px (U/mL)	260.4 ^a^	200.9 ^b^	191.4 ^b^	17.5	0.019
	SOD (U/mL)	71.23	72.68	75.70	4.67	0.788
	MDA (nmol/mL)	8.41 ^a^	6.99 ^b^	8.66 ^a^	0.31	0.004
	TAOC (nmol/mL)	1.51	1.43	1.28	0.09	0.211
Liver	CAT (U/mgprot)	5.60 ^a^	4.20 ^ab^	3.85 ^b^	0.54	0.05
	GSH-Px (U/mgprot)	104.8 ^a^	89.12 ^b^	81.05 ^b^	4.73	0.006
	SOD (U/mgprot)	383.6 ^a^	327.2 ^b^	341.0 ^ab^	15.2	0.036
	MDA (nmol/mgprot)	0.35 ^b^	0.65 ^a^	0.73 ^a^	0.05	0.0002
	TAOC(nmol/mgprot)	0.080 ^b^	0.100 ^a^	0.095 ^ab^	0.005	0.05
Breast Muscle	CAT (U/mgprot)	0.76 ^a^	0.69 ^ab^	0.54 ^b^	0.05	0.03
	GSH-Px (U/mgprot)	16.44	14.01	14.54	0.75	0.071
	SOD (U/mgprot)	104.50 ^a^	78.16 ^b^	84.89 ^b^	3.83	0.0003
	MDA (nmol/mgprot)	2.17	1.76	1.82	0.15	0.128
	TAOC (nmol/mgprot)	0.160 ^a^	0.130 ^b^	0.080 ^c^	0.009	<0.0001
		**Phase II (42 d)**				
Serum	CAT (U/mL)	0.60 ^b^	0.58 ^b^	0.81 ^a^	0.04	0.001
	GSH-Px (U/mL)	275.0	315.6	276.6	18.9	0.266
	SOD (U/mL)	80.5	85.3	75.4	4.7	0.381
	MDA (nmol/mL)	8.09	7.91	8.72	0.48	0.488
	TAOC (nmol/mL)	1.47 ^a^	1.03 ^b^	1.23 ^ab^	0.09	0.011
Liver	CAT (U/mgprot)	3.45	3.37	3.07	0.29	0.619
	GSH-Px (U/mgprot)	83.18	73.47	73.39	3.75	0.114
	SOD (U/mgprot)	314.0	308.6	350.7	16.4	0.19
	MDA (nmol/mgprot)	0.63	0.80	0.72	0.06	0.151
	TAOC (nmol/mgprot)	0.103	0.090	0.111	0.007	0.138
Breast Muscle	CAT (U/mgprot)	0.85 ^a^	0.60 ^b^	0.62 ^b^	0.06	0.018
	GSH-Px (U/mgprot)	10.58	10.53	8.30	0.74	0.078
	SOD (U/mgprot)	84.2 ^b^	85.3 ^b^	98.0 ^a^	3.0	0.009
	MDA (nmol/mgprot)	1.54	1.35	1.39	0.09	0.277
	TAOC (nmol/mgprot)	0.076	0.077	0.076	0.002	0.967

^1^ Normal corn diet (NC); low aging corn (ACL); high aging corn (ACH). ^2^ Catalase (CAT), superoxide dismutase (SOD), glutathione peroxidase (GSH-Px), malondialdehyde (MDA), antioxidative capability (T-AOC). The data are presented as the mean of *n* = 6 for each group. ^a–c^ Mean within each row with different superscripts differ significantly (*p* < 0.05).

**Table 7:** Effect of different levels of aging corn on blood lipid and liver enzymes of broilers fed three different experimental diets ^1^.

Measurements ^2^	NC	ACL	ACH	SEM	*p*-Value
Phase 1					
ALT (U/L)	6.38	6.33	5.50	0.86	0.732
AST (U/L)	239.1	213.8	276.7	22.0	0.181
TC (nmol/L)	3.50 ^a^	2.42 ^b^	3.61 ^a^	0.22	0.003
TG (nmol/L)	0.43 ^ab^	0.37 ^b^	0.47 ^a^	0.02	0.033
HDL-C (nmol/L)	2.02 ^a^	1.35 ^b^	2.10 ^a^	0.14	0.004
LDL-C (nmol/L)	0.44 ^ab^	0.35 ^b^	0.53 ^a^	0.04	0.031
Phase 2					
ALT (U/L)	14.88	11.17	17.33	3.53	0.507
AST (U/L)	713.5	403.8	664.5	122.5	0.193
TC (nmol/L)	3.29	2.77	3.06	0.17	0.113
TG (nmol/L)	0.41	0.41	0.39	0.04	0.94
HDL-C (nmol/L)	1.71	1.51	1.53	0.08	0.17
LDL-C (nmol/L)	0.59	0.40	0.56	0.07	0.147

^1^ Normal corn diet (NC); low aging corn (ACL); high aging corn (ACH). ^2^ Level of serum alanine aminotransferase (ALT); level of serum aspartate aminotransferase (AST); Level of serum total cholesterol (TC); level of serum total triglyceride (TG); level of serum high-density lipoprotein (HDL-C); and level of serum low-density lipoprotein (LDL-C). The data are presented as the mean of *n* = 6 for each group. ^a,b^ Mean within each row with different superscripts differ significantly (*p* < 0.05).

**Table 8:** Effect of different levels of aging corn on jejunum morphology and mRNA gene expression in broilers fed three different experimental diets ^1^.

Measurment ^2^	NC	ACL	ACH	SEM	*p*-value
	Phase 1				
Jejunal Morphology					
VH (µm)	943.3 ^b^	1086.5 ^b^	1406.1 ^a^	58.9	<0.0001
CD (µm)	182.7	173.5	202.7	16.6	0.462
VH/CD	5.34 ^b^	6.55 ^ab^	7.20 ^a^	0.50	0.048
GC (×10^−3^)/µm^2^	3.56 ^a^	2.13 ^b^	2.69 ^ab^	0.33	0.017
Jejunal Tight Junction Proteins					
CLDN1	1.00	0.88	0.69	0.29	0.754
ZO-1	0.99	0.81	0.75	0.14	0.392
OCLN	1.00	0.53	0.49	0.25	0.266
	**Phase 2**				
Jejunal Morphology					
VH (µm)	1216.8	1273.5	1211.6	83.4	0.845
CD (µm)	220.9	272.7	271.1	25.9	0.297
VH/CD	6.12 ^a^	4.76 ^b^	4.55 ^b^	0.43	0.037
GC (×10^−3^)/µm^2^	1.76 ^b^	2.74 ^a^	1.95 ^b^	0.21	0.009
Jejunal Tight Junction Proteins					
CLDN1	1.00	1.27	1.28	0.26	0.654
ZO-1	0.99	1.30	1.18	0.12	0.199
OCLN	1.00	1.12	1.25	0.11	0.319

^1^ Normal corn diet (NC); low aging corn (ACL); high aging corn (ACH). ^2^ VH = villus height; CD = crypt depth; GC = goblet cell; CLDN1 = Claudin1; ZO-1 = Zona Occludin-1; OCLN = Occludin. The data are presented as the mean of *n* = 6 for each group. ^a,b^ Mean within each row with no common superscript differ significantly (*p* < 0.05).

**Table 9:** Effect of different levels of aging corn on volatile fatty acids in ileal and cecal chyme of broilers.

Treatments ^1^	NC	ACL	ACH	SEM	*p*-Value
Ileal VFA ^2^ (µmol/g)		Phase I			
AA	23.82 ^b^	100.10 ^a^	65.14 ^ab^	16.50	0.01
PA	14.79 ^b^	36.31 ^a^	18.22 ^b^	4.39	0.006
BA	15.58 ^b^	73.99 ^a^	49.05 ^a^	8.47	0.0004
		Phase II			
AA	45.56	48.88	68.29	7.63	0.094
PA	27.46	36.76	32.18	4.54	0.355
BA	45.08 ^b^	105.39 ^a^	93.89 ^a^	14.00	0.013
Cecal VFA ^2^ (µmol/g)		Phase I			
AA	63.58	98.33	98.05	16.30	0.22
PA	25.58	37.74	28.33	6.24	0.375
BA	41.00 ^b^	70.51 ^ab^	86.16 ^a^	11.50	0.031
		Phase II			
AA	59.25	94.76	70.99	12.60	0.154
PA	22.61 ^b^	38.69 ^a^	27.61 ^ab^	3.85	0.025
BA	48.90 ^b^	85.86 ^a^	60.47 ^ab^	9.12	0.023

^1^ Normal corn diet (NC); low aging corn (ACL); high aging corn (ACH). ^2^ VFA = volatile fatty acid; AA = acetic acid; PA = propionic acid; BA = butyric acid. The data are presented as the mean of *n* = 6 for each group. ^a,b^ Mean within each row with no common superscript differ significantly (*p* < 0.05).

**Table 10:** Effect of different levels of aging corn on alpha (α) diversity in cecal chyme of broilers.

Treatment ^1^	NC	ACL	ACH	SEM	*p*-Value
Cecal Digesta ^2^			Phase I		
OS	246.2	230.3	288.0	54.2	0.763
SH	4.66	4.36	5.33	0.88	0.753
SI	0.88	0.85	0.89	0.07	0.925
CH	284.3	264.3	314.2	55.4	0.838
ACE	282.8	271.9	314.0	50.9	0.846
GC	0.998	0.998	0.999	0.0002	0.317
PD	16.98	15.72	19.53	2.85	0.671
			Phase II		
OS	699.5	996.8	729.5	93.1	0.075
SH	6.39	6.43	6.73	0.20	0.447
SI	0.96	0.95	0.97	0.01	0.422
CH	755.7	1073.5	794.4	94.6	0.062
ACE	779.9	1078.8	789.2	95.4	0.071
GC	0.998	0.998	0.998	0.0003	0.214
PD	40.72 ^b^	71.07 ^a^	38.04 ^b^	8.65	0.029

^1^ Normal corn diet (NC); low aging corn (ACL); high aging corn (ACH). ^2^ OS = observed species; SH = Shannon; SI = Simpson; CH = Chao1; ACE = abundance-based coverage estimator metric; GC = good coverage; PD = phylogenetic distance. The data are presented as the mean of *n* = 6 for each group. ^a,b^ Mean within each row with no common superscript differ significantly (*p* < 0.05).

## Data Availability

Data are available upon request.
